# DNA damage tolerance branches out toward sister chromatid cohesion

**DOI:** 10.1080/23723556.2015.1035478

**Published:** 2015-05-07

**Authors:** Dana Branzei

**Affiliations:** IFOM, FIRC Institute of Molecular Oncology, Milan, Italy

**Keywords:** Developmental disorders, DNA replication, DNA damage tolerance, mechanisms of oncogenesis and tumor progression, Polα/primase/Ctf4, repriming, replication fork architecture, replication stress, sister chromatid cohesion

## Abstract

Genome duplication is temporarily coordinated with sister chromatid cohesion and DNA damage tolerance. Recently, we found that replication fork-coupled repriming is important for both optimal cohesion and error-free replication by recombination. The mechanism involved has implications for the etiology of replication-based genetic diseases and cancer.

To maintain genome stability, chromosome replication must faithfully preserve genome content in an optimal chromatin structural context. This requirement is met by temporal coordination of DNA replication with DNA damage tolerance (DDT), which promotes completion of replication, and with pathways associated with chromosome structural integrity, such as sister chromatid cohesion (SCC).^1^ Although important functions and key players of these fundamental DNA metabolism processes have been outlined, much less is known about the choreography and mechanistic interplay between SCC and DDT and their contribution to DNA replication. Moreover, the principles by which conserved replisome components uniquely or commonly affect replication-associated chromosome integrity functions remain poorly understood. In a recent study, my group explored the mechanistic basis of this coordination.^2^

Chromosome replication is carried out by the replisome machinery, which is initially assembled at replication origins.^1^ Replication initiation critically depends on the loading and activity of the complex of polymerase α (Polα) and primase, which synthesizes RNA-DNA primers that are subsequently extended by replicative polymerases. Polα/primase-mediated processes are also essential for origin-independent replication initiation events, as in the case of lagging strand DNA synthesis, and potentially for reactivating stalled replication forks.^3^ Polα/primase is tethered and functionally coupled to the replicative helicase mini-chromosome maintenance (MCM) by the conserved chromosome transmission fidelity factor Ctf4,^4^ but this coupling is not essential for genome duplication. We started out by addressing the effect of Polα/primase/Ctf4 mutants proficient in bulk DNA replication on DDT. Two conserved modes of DDT, recombination-mediated (error-free) and mutagenic (error-prone), are used in all eukaryotic cells in response to replication damage.^1^ The error-prone mode involves specialized translesion synthesis polymerases that can replicate across lesions but sometimes cause incorporation of mutations. The error-free mode relies on recombination and a switch of the replicative polymerase from the damaged strand to the undamaged sister chromatid (template switching), and is mediated via the transient formation of recombination intermediates in the rear of the replication fork.^5^ In the article by Fumasoni *et al.*, we report that replicative helicase-coupled repriming is important for efficient error-free replication by template switching, thus preventing the high levels of mutagenesis and faulty strand-annealing events associated with genome rearrangements.^2^ Unexpectedly, repriming defects were also invariably associated with suboptimal SCC^2^ (Fig. 1). But did the coincident defects in recombination-mediated DDT and SCC reflect coordination between these chromosome metabolism processes? This was the next question that we addressed.

**Figure 1.**
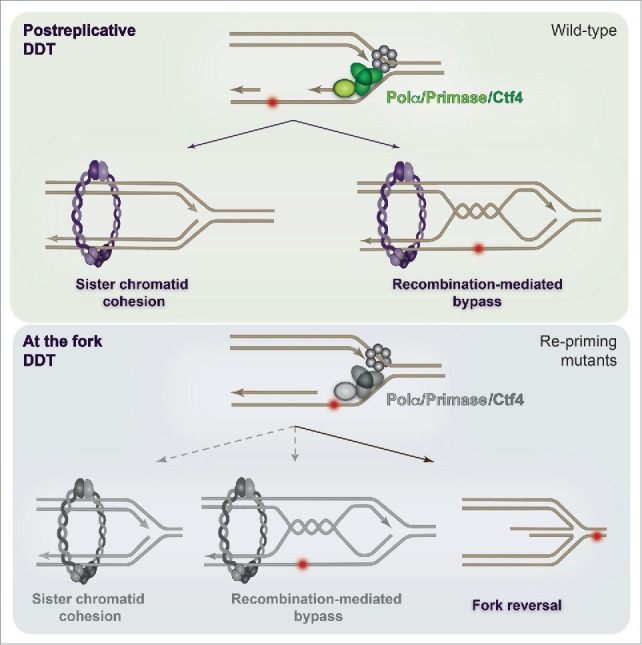
Effects of repriming and DNA damage tolerance (DDT) on replication-associated DNA metabolism. Efficient repriming supports postreplicative error-free DDT and cohesin-mediated functions in sister chromatid cohesion (SCC) and recombination (upper panel). Defective repriming causes an altered pattern of single-strand (ss) DNA stretches at the fork, as well as internal gaps (upper panel). This causes a shift in the location of DDT with respect to the replication fork, and, as a consequence, affects replication fork architecture and the choice of DDT pathway employed. The observed negative effects on SCC likely reflect the complex interplay between defective ssDNA metabolism and altered replication fork architecture.

Cohesin is essential for SCC establishment during DNA replication.^6^ Moreover, when double-strand breaks (DSBs) that threaten genome integrity form postreplicatively, cohesin acts as a splint to direct repair of the broken mitotic chromosome toward the sister chromatid template.^6^ Much less is known about the roles of cohesin and other cohesion factors in DDT processes, which primarily operate on single-stranded (ss) DNA. We revealed that both cohesin and Polα/primase/Ctf4 support template switching, but their roles are fundamentally different: whereas artificial cohesion (experimentally induced by protein bridge-mediated sister chromatid tethering) bypasses the local template switch defects of cohesin mutants, it does not rescue mutants of Polα/primase/Ctf4 or other recombination mutants.^2^ In a nutshell, these results imply that cohesin plays a structural role in aiding postreplicative recombination-mediated DDT by keeping the sister chromatids together. In contrast to cohesin, the SCC and template switch defects associated with Polα/primase/Ctf4 mutants are non-causal and, as we further uncovered in our work using genetic and molecular readouts, derive from altered ssDNA metabolism. What is the significance of these results for our understanding of replication-associated processes or replication-based human disorders?

First, our study indicates that error-free replication following genotoxic stress is greatly influenced by replication fork-coupled repriming. When this repriming function is compromised, DNA metabolism events that are kinetically disfavored in wild-type cells and involve fork reversal and genome rearrangements become more frequent (Fig. 1). The relationship between fork reversal and faulty strand annealing-mediated genome rearrangements is likely to be complex: both can be independently triggered by prolonged pausing and accumulation of ssDNA at the fork; moreover, processing or failed restart of reversed forks may cause breaks and induce recombination. As the replication steps of the triggered recombination pathways will be similarly defective, increasingly more replication stress and deleterious substrates would be created, driving new cycles of genome instability. Thus, our recent results also provide indirect evidence for a replication-based mechanism coupled with defective error-free DDT as an important factor in the etiology of the complex genomic rearrangements found in many cancers and human genomic disorders.^7^

The repriming conditions that we recently described are not only associated with defective error-free DDT and altered replication fork architecture, but also negatively influence SCC^2^ (Fig. 1). In an interesting parallel, hypomorphic mutations in cohesin, replication initiators, and DNA damage response/tolerance factors implicated in ssDNA processing have been reported as driver alleles in clinically similar developmental disorders, such as cohesinopathies, Meier-Gorlin, Seckel, and Jawad disorders.^8-10^ However, any commonality in the underlying mechanisms remained elusive. Our recent work on repriming revealed an interesting intersection between these replication-associated processes, outlining common cues that may explain the similar phenotypes of these diseases. We thus propose a common replication-associated DDT defect that can lead to alterations in fork architecture and sister chromatid proximity as an underlying source of chromosome lesions in a number of replication-based developmental disorders. The knowledge derived from this work and the newly emerging questions point the way to new studies with the potential to reveal the coordination between damage tolerance and chromatin structure functions that are important for the preservation of genome integrity.

## Disclosure of Potential Conflicts of Interest

No potential conflicts of interest were disclosed.
